# Post-acute sequelae of SARS-CoV-2 infection in health care workers from South Africa

**DOI:** 10.1093/oxfimm/iqae001

**Published:** 2024-03-09

**Authors:** Sthembile Mbotwe-Sibanda, Gaurav Kwatra, Shabir A Madhi, Marta C Nunes

**Affiliations:** South African Medical Research Council, Vaccines and Infectious Diseases Analytics Research Unit, Faculty of Health Sciences, University of the Witwatersrand, Johannesburg 2050, South Africa; South African Medical Research Council, Vaccines and Infectious Diseases Analytics Research Unit, Faculty of Health Sciences, University of the Witwatersrand, Johannesburg 2050, South Africa; Division of Infectious Diseases, Department of Pediatrics, Cincinnati Children's Hospital Medical Center and University of Cincinnati, Cincinnati, OH 45229-3026, United States; Department of Clinical Microbiology, Christian Medical College, Vellore 632002, India; South African Medical Research Council, Vaccines and Infectious Diseases Analytics Research Unit, Faculty of Health Sciences, University of the Witwatersrand, Johannesburg 2050, South Africa; Wits Infectious Diseases and Oncology Research Institute, Faculty of Health Sciences, University of the Witwatersrand, Johannesburg 2050, South Africa; South African Medical Research Council, Vaccines and Infectious Diseases Analytics Research Unit, Faculty of Health Sciences, University of the Witwatersrand, Johannesburg 2050, South Africa; Center of Excellence in Respiratory Pathogens (CERP), Hospices Civils de Lyon (HCL) and Centre International de Recherche en Infectiologie (CIRI), Équipe Santé Publique, Epidémiologie et Ecologie Evolutive des Maladies Infectieuses (PHE3ID), Inserm U1111, CNRS UMR5308, ENS de Lyon, Université Claude Bernard—Lyon 1, Lyon 6900, France

**Keywords:** health care workers, post-acute sequelae of SARS-CoV-2, COVID-19 symptoms

## Abstract

Health care workers (HCWs) are primary health providers therefore ensuring their protection and recovery from Covid-19 is of high interest. We investigated post-acute sequelae of SARS-CoV-2 infection (PASC) in HCWs who had previously been infected with SARS-CoV-2. Overall, 68 HCWs were classified as PASC according to duration of persisting symptoms. The 68 HCWs with PASC were split into two groups according to the mean duration of their symptoms, which were (8 PASC) 122 and (60 PASC) 641 days. The frequencies of common symptoms reported by HWCs with PASC were continuous headaches (45), mild cough (41), fatigue (37), myalgia (25) and shortness of breath (14). When using the Medical Research Council (MRC) dyspnoea scale to examine the degree of breathlessness in relations to activity we found that 4 reported having difficulty breathing after strenuous exercise, 19 were identified with shortness of breath when walking fast or when walking up a slight hill, 2 reported walking slower than most people on level or stopping after 15 minutes walking at own pace, 1 reported stopping to breath after walking 91 meters, or after a few minutes on level ground and 1 reported being too breathless to leave the house, or breathless when dressing/undressing. Our results highlight concern for HCWs with long-term persisting symptoms which may negatively impact their health this represents an emerging public health priority. HCWs with prolonged Covid-19 symptoms especially breathing difficulties need better diagnostic tests and treatments.

## Introduction

According to the World Health Organization (WHO), post-acute sequelae of SARS-CoV-2 infection (PASC) happens in individuals with a history of probable or confirmed SARS-CoV-2 infection usually 91 days from the onset of coronavirus disease (Covid-19) with symptoms that last for at least 61 days and cannot be explained by an alternative diagnosis [1]. PASC refers to a wide range of new, returning, or ongoing symptoms in people who have had Covid-19. Although PASC is being increasingly reported in the global north, there remains limited data emerging from low and middle-income countries. A mounting body of evidence has underscored the prominence of PASC cases since the early stages of the Covid-19 pandemic [2, 3]. In South Africa, the declaration of a National State of Disaster was implemented in March 2020 [4]. The long-term effects of Covid-19, even after the conventional “recovery phase” of 14 days, have evolved into a substantial burden [5–7]. PASC cases have predominantly been documented among patients who experienced severe SARS-CoV-2 infections and/or were hospitalized during the acute phase [8, 9]. It has, however, been reported that asymptomatic and mild Covid-19 symptomatic individuals also developed PASC [10–13].

Considering that frontline health care workers (HCWs) constituted a high-risk group for SARS-CoV-2 infection, their active surveillance could prove instrumental in the early, and timely identification of SARS-CoV-2 infections, along with the comprehensive documentation of Covid-19 symptoms spanning from the onset of infection to as long as they persist.

The aim of this this study was to investigate the frequency of PASC in HCWs. We assessed the common Covid-19 symptoms experienced by HCWs, including their duration, furthermore, we employed a dyspnoea scale to gauge the extent of breathlessness experienced by HCWs in relation to their activities.

## Materials and methods

### Study design and population

The current PASC study was a sub-study within the framework of a longitudinal surveillance cohort involving HCWs at the Chris Hani Baragwanath Academic Hospital (CHBAH) in South Africa. The longitudinal study unfolded in two distinct enrolment phases: one from April to July 2020 and another from February to August 2021, details have been previously published [14, 15]. Within this study, we categorized mild Covid-19 episodes as those in which symptoms did not require hospital admission for treatment. PASC cases were defined as those HCWs with a previously polymerase chain reaction (PCR)-confirmed SARS-CoV-2 infection and experiencing Covid-19 symptoms for more than 61 days after the initial infection.

All HCWs completing the longitudinal surveillance study were given two questionnaires at the end of the study in April 2022. These questionnaires were thoughtfully designed to document prevalent symptoms and their duration, identify participants experiencing PASC, and identify any persistent symptoms among the PASC population. Participants were requested to record the exact number of days their symptoms persisted, the duration of symptoms were categorized per days reported. Additionally, the questionnaires incorporated the Medical Research Council (MRC) dyspnoea scale to investigate the state of breathlessness in relation to activity. This tool developed by the Oxford Academy and the United Kingdom Research and Innovation Centre included five statements that comprehensively depict the spectrum of respiratory impairment, ranging from none to nearly complete incapacity [16, 17].

Participants were asked to select the phrase that most accurately encapsulated their current condition or during the time symptoms relay from first infection. The questionnaires were self-administered, and participants were able to answer all questions digitally with the assistance from trained staff members.

### Data collection and presentation

HCWs were initially enrolled in the longitudinal cohort and were requested to visit the study clinic every 1 to 2 weeks for PCR testing, irrespective of symptoms suggestive of Covid-19, if they tested positive for SARS-CoV-2 the scheduled visits continued.

The eligibility criteria to be included in the present analysis were that HCWs must have completed the end-of-study questionnaires, tested positive for SARS-CoV-2 by PCR and experienced Covid-19 symptoms persisting after their initial positive PCR test. In case of re-infections, participants were instructed to report symptoms from their first SARS-CoV-2 positive test.

SARS-CoV-2 infections were detected among HCWs participating in this PASC study between June 10, 2020 and January 22, 2022. At the conclusion of the main HCW cohort study in April 28, 2022, all PASC participants had surpassed a minimum of 91 days post their SARS-CoV-2 infections. We report the frequency of Covid-19 symptoms persisting for a minimum of 91 days and longer.

The four most common SARS-CoV-2 variants of concern circulating in South Africa were used to infer the variants that infected the study population taking into account the time periods that infections occurred [18]. As such, the ancestral Wuhan virus caused the first wave from March, 2020 to November, 2020; the second wave from November 20, 2020 to mid-May 2021 was dominated by the Beta variant; the Delta variant caused the third wave from mid-May 2021 to mid-November 2021, and the Omicron BA.1/BA.2 variant was responsible for the fourth wave that started in mid-November 2021 and lasted to March 2022.

Categorical variables are summarized as frequencies, and continuous variables are expressed as median and interquartile range (IQR) or mean and standard deviation. Analyses were performed using Stata/SE 17 software. All study data was recorded on the Research Electronic Data Capture (Redcap) platform. The sub-study was approved by the University of Witwatersrand Human Research Ethics Committee (Medical) (Approval Number: 220710).

## Results

In the longitudinal cohort 549 HCWs were enrolled. At the end of study 104 (18.9%) HCWs who had tested PCR positive for SARS-CoV-2 completed the last visit questionnaires. Of these 68 (65.4%) reported symptoms lasting longer than 91 days and 36 (34.6%) reported mild symptoms that subsided within 30 days and not longer. Results of the F-test for equality of variances indicated that there was a non-significant difference between Female (SD1 = 8.9) and Male (SD2 = 6.5), F(91,11) = 1.9, *P* = 0.248. At the time that the last visit questionnaires were answered 379 median days (range: 45—680 days) had passed from initial infection, Table 1 summarizes the characteristics of the previously infected HCWs who completed the questionnaires, stratified by when the symptoms resolved.

**Table 1.** Characteristics of participants previously infected with SARS-CoV-2

	Overall *N* = 104	Symptoms lasting 91–183 days *N* = 8 PASC	Symptoms lasting 213–365 days *N* = 60 PASC	Symptoms no longer than 30 days *N* = 36
Female, *n* (%)	92 (88.5)	6 (75)	55 (91.7)	29 (80.6)
Male, *n* (%)	12 (11.5)	2 (25)	5 (8.3)	7 (19.4)
Female Mean age in years (standard deviation)	45 (8.9)	52.5 (6.5)	45 (8.9)	44 (9.1)
Male Mean age in years (standard deviation)	47 (9.8)	49.5 (10.5)	42 (10.7)	45.6 (9.5)
Median days between infection and questionnaire completion, min.-max, interquartile range	379, 45–680, 142–642	122, 94 - 189, 108–137	641, 235–680, 541–655	140.5, 45–644, 122–246

**Figure 1.**
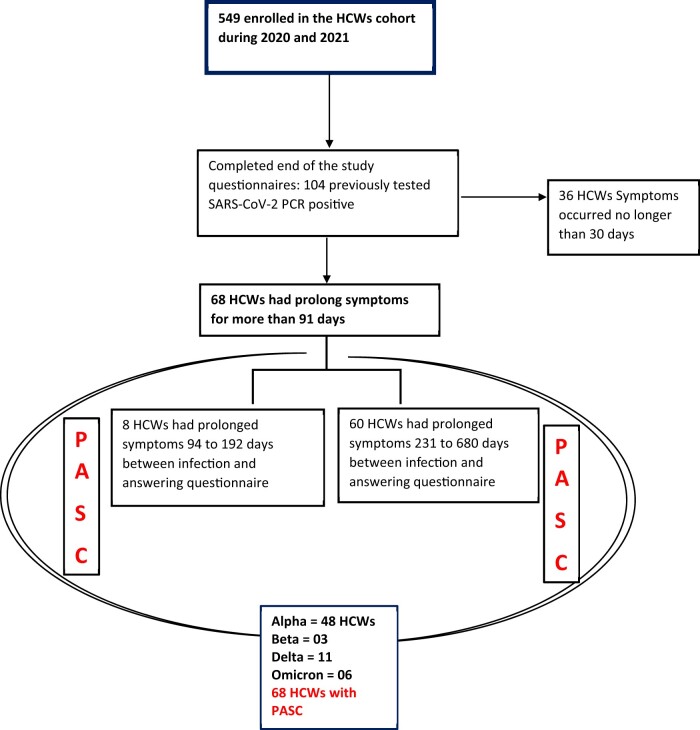
Study population based on inclusion criteria. HCWs- Health Care Workers, PASC- Post-acute sequelae of SARS-CoV-2

Infections occurred during the four Covid-19 pandemic waves. We looked closely at the HCWs with PASC, a total of 48 were infected during the first wave, when the ancestral Wuhan virus was circulating, three HCWs were infected during the Beta wave, 11 were infected during the Delta wave and six were infected during the Omicron wave (Fig. 1).

**Figure 2.**
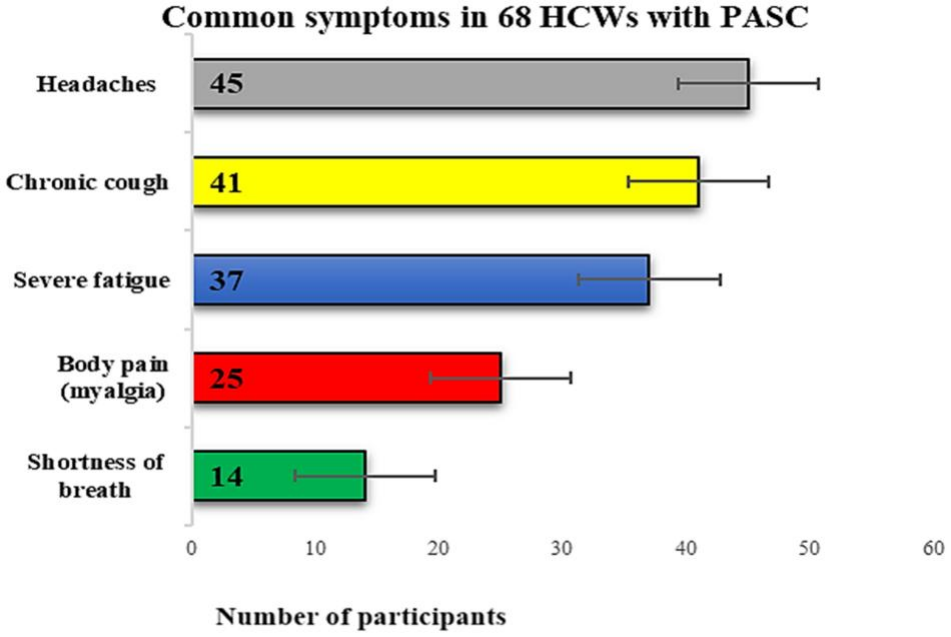
Frequencies of common symptoms reported by the 68 HCWs with PASC. Each bar represent the frequencies of the symptoms with error bars. The number of participants with symptoms is indicated in each bar

Next, we identified the most common reported symptoms among the 68 HCWs with PASC. The frequencies of common symptoms reported were: continuous headaches (45), mild cough (41), fatigue (37), myalgia (25) and shortness of breath (14) (Fig. 2).

### MRC dyspnoea scale grade degree of breathlessness related to activity

Overall 104 HCWs completed the last visit questionnaires, and the 68 HCWs classified as PASC completed the MRC dyspnoea scale (Table 2). Among these 41 (60.3%) reported that breathing was not affected after the recovery phase, 4 (5.9%) reported having difficulty breathing after strenuous exercise (grade 1), 19 (27.9%) were identified with shortness of breath when walking fast or when walking up a slight hill (grade 2), 2 (3.0%) reported walking slower than most people on level or stopping after 15 minutes walking at own pace (grade 3), 1 (1.5%) reported stopping to breath after walking 91 meters, or after a few minutes on level ground (grade 4) and 1 (1.5%) reported being too breathless to leave the house, or breathless when dressing/undressing (grade 5).

**Table 2.** MRC dyspnoea scale Grade Degree of breathlessness related to activity

Grade	Degree of breathlessness related to activity per grade	Number of HCWs (percentage)
1	Breathless/difficulty breathing after strenuous exercise	4 (5.9%)
2	Short of breath when walking fast or when walking up a slight hill	19 (27.9%)
3	Walks slower than most people on level or stops after 15 min walking at own pace	2 (3.0%)
4	Stops to breathe after walking 91 m, or after a few minutes on level ground	1 (1.5%)
5	Too breathless to leave the house, or breathless when dressing/undressing	1 (1.5%)

Adapted from Fletcher^42^.

## Discussion

We report that persisting mild Covid-19 symptoms occurred for longer than 94 days post infection among 68 HCWs with confirmed SARS-CoV-2 infection. To our knowledge, this is the first descriptive study on PASC amongst HCWs in South Africa with mild Covid-19 symptoms.

In recent studies it has been shown that individuals with mild Covid-19 symptoms for less than 30 days reported higher frequency of acute symptoms such as cough, fever, sleep disturbances and headaches [13, 19–21] whereas studies with follow-up beyond 61 days reported fatigue, chronic coughs, and musculoskeletal symptoms more frequently [22–25]. In our study, shortness of breath was the least reported symptom by HCWs with PASC. However, when we used the MRC dyspnoea scale, breathing problems were present in the HCWs with PASC, an increased number of HCWs had shortness of breath when hurrying on a level ground or when walking up a slight hill.

It has been previously reported that risk factors for developing PASC among individuals with mild Covid-19 symptoms, non-hospitalized, were age and gender [26–29]. With older adults ≥ 65 years having an increased risk of new or worsening disability and new or persistent symptoms [25, 30–32]. This association might result from increased comorbid disease, poorer overall health status, and relative exertion intolerance [31, 33]. There is conflicting evidence in the literature on age as a risk factor for PASC. Some studies found an association between age younger than 65 years and persistence of symptoms [34], and with participants of younger age being are more likely to survive acute infection, thereafter have worse long-term effects [35, 36]. We found no association between age and development of PASC, this could be due to majority of the HCWs who participated in the study were in their mid-forties to early fifty age group. Being female has been found to have a significant association with PASC in various studies [27, 28, 35], including association with fatigue and breathlessness [17, 28]. In our study, results of the F-test for equality of variances indicated that there was no difference between Female and Male *P* = 0.248. The health-seeking behaviour in men is lower than in women, and men might be less enthusiastic to report persistence of bad health [32].

To understand the impact of PASC, infections should be reported in terms of the circulating SARS-CoV-2 strains or the pandemic waves [4, 18, 36]. The similarities between symptoms was not seen across the different variants. However, severity of sequelae following infection from the first and second SARS-CoV-2 waves were greater due to infections in the study that were detected during the first wave. The possible decrease in infections in the third and fourth wave could have been due to the vaccine roll-out in South Africa which started in February 2021. Initially the Ad26.COV2.S Covid-19 vaccine was first given to HCWs [4, 15, 36, 37]. In clinical trials, a single-dose of Ad26.COV2.S vaccine demonstrated 66% and more effectiveness overall in preventing moderate and severe Covid-19 disease, 28 days post-vaccination [38–41]. We have previously shown that there was high re-infection rate and vaccine breakthrough infection rates with the Omicron SARS-CoV-2 variant among these HCWs however severity of infection tended to be milder compared with other waves [15].

WHO clarified that PACS symptoms might be of a new onset after initial recovery from an acute Covid-19 episode or they may persist from the initial illness, also these symptoms might fluctuate and relapse over time [1]. This reveals knowledge gaps regarding re-infections in individuals with PASC and the effect that re-infections have on the ongoing persisting symptoms.

### Limitations of this study

There were not enough participants to determine statistical differences for all common symptoms reported in the PASC survey.

## Conclusions

Attention needs to be given to HCWs with prolonged mild Covid-19 symptoms, especially breathing difficulties. Better treatments and diagnosis tools are needed to treat PASC which will ease the physical burden and stresses in HCWs.

## Data Availability

Data was collected on study-specific data collection forms and entered on a specially designed Redcap database. Data was entered and stored on a secure online research electronic data capture repository (REDCap, version 10.6.14, University of the Witwatersrand). Data management was centralized at Wits VIDA. This system enables real-time data entry, real-time monitoring of data, generation of weekly reports, and timeous dissemination of results.

## Data availability

The data underlying this article will be shared on reasonable request to the corresponding author.
